# The Manipulation and Care of Cements

**Published:** 1903-04-15

**Authors:** W. V-B. Ames

**Affiliations:** Chicago, Illinois


### THE MANIPULATION AND CARE OF CEMENTS.

BY W. V-B. AMES, D.D.S., CHICAGO, ILLINOIS.

A phosphoric acid solution, pure and simple, in a proper degree of concentration, will so combine with certain metallic oxids, notably zinc oxid, as to make a workable cement, if proper conditions are observed. While the liquids of cements of this kind in use in dental practice are never simple solutions of phosphoric acid, the same requisites of manipulation hold good to a considerable extent. To be sure, some of the acid solutions largely neutralized with alkaline phosphates need very little consideration in the mix to obtain the best results possible to the combination; but with what I will call the better class of cements, those in which the non-alkaline metallic phosphates have been used to modify the working qualities of the acid, certain precautions are necessary, which can be best illustrated by considering a mix of simple syrupy phosphoric acid and basic zinc oxid.

### TECHNIQUE OF MIXING.

If basic zinc oxid is mixed with a concentrated solution of phosphoric acid by making small additions of the oxid and spatulating thoroughly, especially the first additions, a workable mass is obtained which will make a quite satisfactory filling. If, however, the zinc oxid is added in larger portions, without the precaution of spatulating thoroughly the first additions, the mix will set quickly, be granular in packing, and probably generate heat during setting. The difference illustrated here depends simply on the fact that in the gradual mix with much spatulation there is developed a considerable amount of normal phosphate of zinc, from solution of zinc oxid in the acid, which has a modifying influence on the setting process. In the cements referred to as the better class, having a liquid consisting of phosphoric acid modified by such metallic phosphates as the zinc phosphate of our illustration, there is a limitation in the process of treating the acid with the phosphates, as the acid solution will not hold, through all the vicissitudes to which it is apt to be subjected, a proportion of these non-alkaline metallic phosphates sufficient for ideal working qualities.

We will suppose or assert that in this class of cements the liquid has been treated with non-alkaline metallic phosphates to the limit of safety, beyond which there would be a probability of crystallization—which is always detrimental if not disastrous. Because of this limitation in the compounding of a high-grade cement, it becomes necessary in their manipulation to adopt to an extent the procedures described in connection with the plain ortho-phosphoric acid, which will usually give the difference, especially in midsummer, between smooth plasticity with desirable setting qualities and a granular consistence with objectionably quick setting.

My incentive for the making of these points has been from very frequently seeing the ingredients of a mix of cement fairly *slopped* together, there being a semi-mixed borderland at the edge of the supply of powder, giving a clotty condition as it is brought into the mix. If the supply of powder is sufficiently removed from that of the liquid and the additions of powder are carried over in a cleanly manner and then thoroughly spatulated, this clotty condition can be entirely avoided.

### PRECAUTIONS AS TO THE LIQUID.

The tendency to crystallization of a properly modified solution of phosphoric acid calls for much care in its handling during consumption. After trying the ordinary cork, the rubber and the glass stopper, also metal screw-cap, I became satisfied that, however the bottle is originally corked, the liquid should never be used from that bottle, but should be transferred to one of proper size having a telescoping glass cap instead of a cork fitting within the neck. The best of the sort obtainable is, I think, the S. S. W. No. 6 office preparation bottle. With the ordinary container, with ordinary handling, there will always be more or less liquid standing about the cork, exposed to the air, which will attract water from the atmosphere during summer months and in winter give up water to the atmosphere of an artificially-heated room. In summer the diluted liquid passes into the bottle on each removal of a cork, altering the specific gravity to that extent, and in winter a crystal from about the cork may start crystallization in the main mass of liquid, which would probably have remained free of crystals if transferred to the other form of bottle, with which this could not happen. The portions of liquid for use may well be removed by means of the *clean* non-corrosivespatula which should be used for the mix. Of non-corrosive spatulas the most practical seems to be that made from a high-grade German silver, the so-called "platinoid" and white metals being apparently of this order. Platino-iridium is probably the very best combination for the purpose, and if socketed into some other metal need not be especially expensive. For a mix of cement in which a slight modification of color is not objectionable a spatula of coin silver or of an alloy of silver and copper containing the proportions giving the maximum rigidity and hardness would be beneficial from a chemical point, supposing that a slight abrasion and chemical action on the metal occur. Phosphates of these metals have a very salutary effect, whereas phosphate of iron, which is necessarily formed to a certain extent when a steel spatula is used, is very detrimental.

The mention of cleanliness in every procedure connected with a mix of cement ought not to be called for, yet since recently seeing a dentist, who has mixed cement long enough to know better, showing at a clinic his so-called new hydraulic and impervious cement and making one mix after another without cleaning his spatula and slab, it seems that a few words on this will not be amiss. The very fact that one crystal of a kind will hasten the formation of other crystals is sufficient reason for beginning a mix of cement with an immaculately-clean spatula and slab.

The time I have allotted myself will not admit of any consideration of the real use of the cements. I only with to refer to the trend in the direction of hydraulic cements for several years, and to predict that this quality, properly developed, will come to be a requisite.—*Cosmos.*

